# Fear and the internalization of external regulation – An exploratory study on how fear of COVID-19 affected the internalization of mask-wearing

**DOI:** 10.1371/journal.pone.0347772

**Published:** 2026-05-12

**Authors:** Jonas Tögel, Christof Kuhbandner

**Affiliations:** Department of Human Sciences, University of Regensburg, Regensburg, Germany; Utah State University, UNITED STATES OF AMERICA

## Abstract

The internalization of externally regulated behavior is a key topic in motivational psychology. While fulfilling the basic psychological needs for autonomy, competence, and relatedness provides the foundation for internalization, less is known about factors that determine its depth. This study examined the role of fear in shaping the internalization of externally regulated behavior, using mask wearing during the COVID-19 pandemic as a real-world example. The continued use of masks after mandate removal provided a natural context to examine how fear influences internalization, as the goal of mask wearing, preventing COVID-19 infection, was potentially associated with strong fear. Following the end of the mandate, participants (*N* = 445) were presented with an everyday scenario and asked whether they would wear a mask. Their motivational states across different types of internal regulation, fear of COVID-19, and beliefs about the effectiveness and side effects of mask-wearing were assessed. Analyses showed that higher levels of fear of COVID-19 were strongly associated with continued mask-wearing. Each one-point increase in fear corresponded to a 2.5-fold increase in the odds of voluntarily maintaining mask use. The likelihood of continued mask-wearing increased with stronger beliefs in mask effectiveness and decreased with higher perceived side effects; however, the effect of fear was independent of these rational beliefs and persisted even among individuals who perceived masks as less effective or with stronger side effects. Regarding the depth of internalization, fear of COVID-19 correlated positively with introjected and identified regulation, but not with integrated or intrinsic regulation. These results indicate that fear can facilitate the internalization of externally regulated behavior independently of rational beliefs about the effectiveness and side effects of the behavior, yet only up to a moderate level of self-determination.

## Introduction

A central goal of motivational psychology is to understand how and under what conditions individuals internalize externally regulated behavior so that it is maintained even in the absence of external contingencies. Although the gradual transition from external to increasingly self-determined forms of regulation has been well described theoretically [1], the factors that determine—beyond the satisfaction of the basic psychological needs for autonomy, competence, and relatedness as general prerequisites for internalization— which level of internal regulation is reached remain insufficiently understood.

### 1.1. The levels of internal regulation

Within Self-Determination Theory (SDT), the internalization of external regulation is described as a process through which behavior initially governed by external contingencies, such as rewards or punishments administered by others, becomes progressively regulated by more self-determined forms of motivation. At the most basic level, behavior may become habitual: conditioned stimulus–response associations are acquired through repeated experiences of externally reinforced reward and punishment. At this stage, behavior is elicited automatically by contextual cues without conscious representation of behavior–outcome relationships [2–4].

At higher levels of internalization, behavior that was initially externally regulated becomes increasingly autonomous and integrated into an individual’s personal value system and sense of self. According to Organismic Integration Theory [1,5], a subtheory within SDT, this process unfolds through several distinct forms of behavioral regulation that differ in their degree of self-determination.

The first step beyond external regulation is introjected regulation, in which a behavior is enacted to avoid negative emotions (e.g., guilt, shame, fear) or to attain positive emotions (e.g., relief, pride). These affective contingencies represent internalized versions of previous external rewards and punishments. Thus, compliance no longer depends on external enforcement, as emotional consequences now maintain the behavior.

The next step, identified regulation, occurs when individuals consciously accept the rationale for a behavior and endorse it as personally meaningful. From a control-value perspective [6], this requires two beliefs: (a) that the goal underlying the behavior is personally valued and worthwhile, and (b) that the behavior is perceived as an effective means of achieving that goal. External contingencies are no longer required, as the individual has personally endorsed the reasons for acting.

A behavior regulated through identification may not necessarily align with other personal values or intrinsically motivated behaviors, which can lead to inner conflict or defensiveness. The subsequent stage, integrated regulation, is reached when the identified behavior is brought into harmony with one’s broader value system and self-concept. The more a behavior becomes integrated, the fewer internal conflicts individuals experience in enacting it.

Finally, intrinsic regulation represents the most self-determined form of motivation. Here, behavior is performed for its inherent satisfaction because it is experienced as interesting or enjoyable in itself [7]. According to SDT, behavior that originally arose solely from external contingencies rarely becomes intrinsically motivated, as its function remains instrumental.

#### Factors influencing the depth of internalization.

Although extensive research has demonstrated that satisfaction of the basic psychological needs for autonomy, competence, and relatedness provides the foundation for internalization, less is known about the factors that determine the extent to which external contingencies are internalized across the different levels of regulation. Some evidence suggests that satisfaction of the needs for competence and relatedness may be sufficient to support introjected regulation, whereas the additional satisfaction of autonomy may be required to foster identified and integrated regulation [8–9]. Moreover, experimental studies have examined more specific influences, such as the type of punishment administered by an external authority [10] or the induction of guilt for noncompliance [11].

From the broader perspective of human motivation, another potential determinant of how deeply external regulation becomes internalized may be the emotional significance of the issue that the externally regulated behavior addresses. The more intense the emotions elicited by the issue, the more likely it may be that the behavior becomes internalized. This influence may be particularly relevant for introjected regulation, where behavior is sustained by internal emotional contingencies, but may play a lesser role in identified and integrated regulation, which are primarily driven by rational and value-based processes [1,7].

A central challenge for this type of research is to create ecologically valid situations that individuals perceive as personally relevant and meaningful, allowing the internalization of external regulation to be studied under realistic conditions. A promising approach in real-life contexts is to compare time periods during which a behavior was externally regulated with subsequent periods in which the external contingencies were removed. Comparing individuals who continue to display the previously externally regulated behavior with those who discontinue it can yield insights into the factors that facilitate or hinder internalization [12]. Furthermore, assessing individuals’ reasons for continuing the behavior allows for identifying the specific levels of internalization that have been achieved.

### 1.2. The internalization of mask-wearing

A unique, ecologically valid opportunity to examine the internalization of external regulation under real-life conditions emerged during the COVID-19 pandemic, when governments in many countries mandated mask-wearing in public [13–15]. In societies where mask-wearing had not previously been customary, such mandates represented a strong form of external regulation. These periods were later followed by phases in which mask-wearing was no longer mandatory but merely recommended, with some individuals continuing to wear masks and others not, making this situation an ideal context for studying the internalization of external regulation. Importantly, the goal underlying mask-wearing—preventing the transmission of SARS-CoV-2—was associated with strong emotional reactions, particularly fear [16–17]. This combination of a clearly defined externally regulated behavior and emotionally charged circumstances provides a highly ecologically valid setting to investigate the role of emotions in the process of internalization [18].

Several studies have already examined adherence to public health measures such as mask-wearing during the COVID-19 pandemic. From a motivational perspective, research has shown that so-called “autonomous motivation”, rooted in personal values, a sense of ownership, and genuine interest, was associated with greater adherence than so called “controlled motivation”, driven by external contingencies or internal pressures such as guilt or shame [19–21]. With regard to fear, previous findings indicate that fear of COVID-19 mediated the association between individuals’ risk perception and their adherence to public health measures [22].

While these studies provide valuable insights into the predictors of compliance, they offer limited information about the conditions under which external regulation becomes internalized. Their focus has been on compliance rather than on the motivational processes underlying internalization, and their conceptualization of motivational states has often been overly broad. Specifically, “controlled motivation” is frequently used as an umbrella term conflating external regulation (behavior guided by external contingencies) and introjected regulation (behavior maintained by internal pressures), whereas “autonomous motivation” is used to encompass multiple levels of more self-determined regulation. Consequently, such studies provide little insight into the factors influencing the transition from externally regulated to more self-determined forms of motivation.

With regard to the different levels of increasingly internalized regulation, the motivational background of mask-wearing can be conceptualized as follows. At the level of acquired habits, mask-wearing may occur as a learned behavioral response resulting from previous mandates and associated legal or social sanctions, enacted automatically without conscious evaluation. At the level of introjection, individuals may wear a mask because it feels appropriate or morally right, whereas not wearing one would evoke discomfort, guilt, or fear of social disapproval. At the level of identification, individuals may endorse mask-wearing because they personally value the goal of protecting health and perceive it as an effective means to achieve this goal. At the level of integration, mask-wearing may be experienced as consistent with broader personal values and self-concept—for example, as an expression of social responsibility or solidarity. Finally, at the level of intrinsic motivation, mask-wearing would have to be experienced as inherently enjoyable or interesting—a theoretically conceivable but empirically rare form of regulation.

### 1.3. The aim of the study

The present study aimed to examine the role of fear in the internalization of external regulation, using the lifting of the mask mandate in Germany as a naturalistic context. Mask-wearing in public became mandatory at the end of April 2020 in Germany, representing a behavior with little prior intrinsic or cultural value. When the mandate was lifted at the beginning of April 2022, individuals were free to decide whether to continue wearing masks. This transition from external to internal regulation provided an ideal naturalistic setting for examining factors that influence internalization.

The data analyzed in this study were drawn from a larger survey conducted after the lifting of the mask mandate, in which participants were presented with a realistic everyday scenario—entering a supermarket where some people were wearing masks—and were asked whether they would currently wear a mask in such a situation. This distinction allowed us to differentiate between individuals who had internalized the previously externally regulated behavior (i.e., those who stated that they would continue to wear a mask) and those who had not (i.e., those who stated that they would not). In addition, participants reported their motivational states across different levels of internal regulation, as well as several other variables, including fear of COVID-19, beliefs about the effectiveness of mask-wearing in preventing SARS-CoV-2 infection, and beliefs about the strength of the side effects associated with mask-wearing. Data on motivational states, fear of COVID-19, and beliefs about mask effectiveness and side effects were analyzed exploratively to provide insights into the role of fear in the internalization of externally regulated behavior.

In a first step, we tested whether individual differences in fear of COVID-19 predicted continued mask-wearing after the mandate had been lifted. If fear facilitates internalization, individuals experiencing higher levels of fear should be more likely to continue wearing a mask. To assess whether fear functions independently of rational beliefs, we examined whether the association between fear and the internalization of mask-wearing varied as a function of participants’ beliefs about mask effectiveness and side effects. If the effect of fear operates independently of rational beliefs, a comparable relationship between the level of experienced fear and continued mask-wearing should be observed regardless of participants’ beliefs about mask effectiveness and side effects.

In a second step, we examined the extent to which the influence of fear extends across the different levels of internal regulation. Participants who reported continuing to wear masks were asked to indicate their reasons for doing so, allowing us to assess their amount of motivation at five levels of internal regulation (extrinsic, habitual, introjected, identified, integrated, and intrinsic motivation). Correlations between fear and these motivational states were then analyzed to determine at which levels fear plays a role in the internalization process. A significant association would suggest that fear contributes to internalization at that specific level, whereas the absence of a correlation would indicate that fear is not a relevant factor at that stage. To examine whether fear functions independently of rational beliefs, correlations between beliefs about the effectiveness and the side effects of mask-wearing and the amount of motivation at the different levels of internal regulation were computed to determine at which levels these beliefs played a role. Subsequently, multiple regression analyses were conducted to explore the interactions between fear and these beliefs at the levels where both fear and rational beliefs were associated with motivation. If fear operates independently of rational beliefs, fear and beliefs about the effectiveness and side effects of mask-wearing should not interact in their effects on the amount of motivation.

## Method

### 2.1. Participants

Data collection started on December 15 2022 and ended on January 31 2023. After the change of the Federal Infection Control Act on March 18 2022, wearing masks was no longer mandatory after April 2 2022 [23]. Participants were recruited primarily through large-scale university lectures and social media platforms. The study did not specifically target university students; rather, a convenience sample of consenting adults was collected to investigate general psychological mechanisms of internalization. Because recruitment primarily occurred in academic contexts, the sample consisted predominantly of university students. No inclusion or exclusion criteria regarding student status were defined. Because the survey was conducted online, participation was not restricted to a specific region; however, since the survey was advertised predominantly at Bavarian universities, most participants lived in Bavaria.

The sample size was determined by including all individuals participating in the study by the end of the data collection period in the data analysis. The final sample consisted of 445 participants. The mean age was 22.2 years (*SD* = 5.1). The sample consisted predominantly of female participants. Detailed demographic characteristics of the sample are presented in Table 1.

**Table 1:** Demographic characteristics of the sample.

Variable	n	M (SD)/ %
Age	—	22.2 (5.1)
Gender		
Female	350	78.7%
Male	91	20.4%
Not specified	4	0.9%
Occupation		
Students	438	96.2%
Employed	16	3.6%
Not specified	1	0.2%

**Note.** Age is reported as mean (SD); all other variables are presented as percentages. N = 445.

The study was conducted in accordance with the Helsinki Declaration and the University Research Ethics Standards of the University of Regensburg. All participants provided informed consent prior to filling out the questionnaire. In Germany, these types of psychological studies do not require ethical approval of an Ethics Committee according to the Deutsche Forschungsgemeinschaft (DFG) [24].

### 2.2. Procedure and measures

The study was conducted online via SoSci Survey [25]. A self-designed questionnaire was available online from December 15, 2022, to January 31, 2023. The questionnaire was constructed deductively based on Organismic Integration Theory (Ryan & Deci, 2000; Ryan & Deci, 2020), as described in the introduction section. For each level of regulation, items were formulated to reflect its defining motivational characteristic: external contingencies (extrinsic regulation), automatic enactment (habitual responding), avoidance of negative affect or self-approval (introjected regulation), conscious valuing and perceived effectiveness (identified regulation), coherence with broader personal values (integrated regulation), and inherent enjoyment (intrinsic regulation). The aim was not scale development but differentiation between regulatory styles; therefore, a small number of conceptually focused items was used for each level. This approach follows common SDT research practice where regulation types are operationalized through theoretically defined motivational reasons rather than psychometric scale construction. The questionnaire was written in German, the native language of the participants. At the beginning of the questionnaire, mask wearing behavior was measured. An everyday situation was described to the participants, and they were asked whether they would wear a mask in this situation or not. The everyday situation was described as follows:

“Imagine you are entering a supermarket to get done with some shopping. Inside there are a few people shopping and the store is well visited, but not crowded. Some of the people are waring face masks, others are not. How would you behave while entering the supermarket? Please check the according box.”

Participants could then select either “I put on a mask.” or “I don’t put on a mask.”

The following 12 items addressed the motivational stages behind the mask wearing behavior. The motivational state at each of the levels was assessed by two statements, and the participants were asked to what extent they agreed with each statement using a Likert scale ranging from 1 (“I strongly agree”) to 4 (“I strongly disagree”). Depending on the participants’ response on the mask wearing behavior item, the statements were formulated either in the present tense (participants who stated that they wear a mask) or the past tense (participants who stated that they did not wear a mask).

Extrinsic motivation was measured using the statements “I used to wear a mask, because you might have to pay a fine for not wearing it” and “I wear a mask because other people look at you funny or address you for not wearing it”. Habitual responding was measured using the statements “I put on a mask without further thinking about it” and “Wearing a mask has become a habit for me”. "Introjection was measured using the statements “I’m wearing a mask because otherwise I’d feel uncomfortable”" and “I`m wearing a mask because it gives me a sense of confirmation that I’m doing the right thing”. Identification was measured using the statements “Personally, it is important to me that the rule of wearing a mask is being complied” and “I am convinced that it makes sense to wear a mask to protect myself and others”. Integration was measured using the statements “I am wearing a mask because I am convinced that wearing it is a part of social togetherness” and “I am wearing a mask because I have weighted the pros and cons and am convinced, that I am acting in solidarity and caring by wearing it”. Intrinsic motivation was measured using the statements “I am wearing a mask because it brings me joy to wear it” and “I am wearing a mask because I find it comfortable”. Participants who stated that they did not wear a mask were finally additionally asked to respond to the statement “I have never worn a mask (for health or personal reasons)”.

After that, several variables were measured, including people’s fear of Covid-19 and beliefs about the effectiveness of mask wearing and the perceived side effects of mask-wearing. Fear of COVID-19 was measured using the statement “I am afraid of Covid-19” which was rated on a Likert scale ranging from 1 (I strongly disagree) to 4 (I strongly agree). Similar single-item measurements to assess fear of COVID-19 have been used in previous research [26]. This allowed us to differentiate between four levels of fear of COVID-19: none, low, medium and high. Beliefs about the effectiveness of mask-wearing were assessed with two items, each rated on a ten-point percentage scale ranging from “0–10%” to “91–100%” in 10% intervals: “How well do you think wearing a mask protects you from infection?” and “How well do you think wearing a mask protects others from infection?”. The mean of both ratings was used as an index of perceived mask effectiveness. Beliefs about the strength of side effects associated with mask-wearing were assessed with a five-point Likert item (1 = “It does not have any side effects” to 5 = “It has very strong side effects”). Participants who indicated that masks have negative side effects were asked to specify these in an open-ended follow-up question.

All items were originally formulated in German. Prior to data collection, the wording was reviewed to ensure its comprehensibility in a small informal pretest. Descriptive statistics of the study variables are reported in S1 Table (Panel A).

The present data were derived from a broader questionnaire on mask-wearing behavior that included additional measures (e.g., media consumption, vaccination status, and the ego-identity dimensions of exploration and commitment). Results concerning these additional variables are [27]/ will be presented elsewhere.

### 2.3. Statistical analyses

To examine whether fear of COVID-19 predicted continued mask-wearing, binary logistic regression analyses were conducted with continued mask-wearing (0 = no, 1 = yes) as the dependent variable. Fear of COVID-19 (four-point Likert scale) was treated as a continuous predictor, followed by models additionally including perceived mask effectiveness and perceived side effects as well as their interaction terms with fear. To investigate the depth of internalization, Pearson correlations were computed between fear of COVID-19 and the motivational variables representing the different levels of regulation. Subsequently, linear regression analyses were conducted for levels of regulation in which both fear and rational beliefs were associated with motivation, including interaction terms between fear and beliefs about mask effectiveness.

All continuous predictors were mean-centered prior to computing interaction terms. Mean-centering changes only the reference point of the scale and does not dichotomize the variable Categorical groupings shown in figures were used only for descriptive illustration. For logistic regression analyses, odds ratios with 95% confidence intervals are reported. Model fit was evaluated using −2 log likelihood and Nagelkerke’s R².

## Results

### Fear of COVID-19 and continued mask-wearing

**Fig 1:**
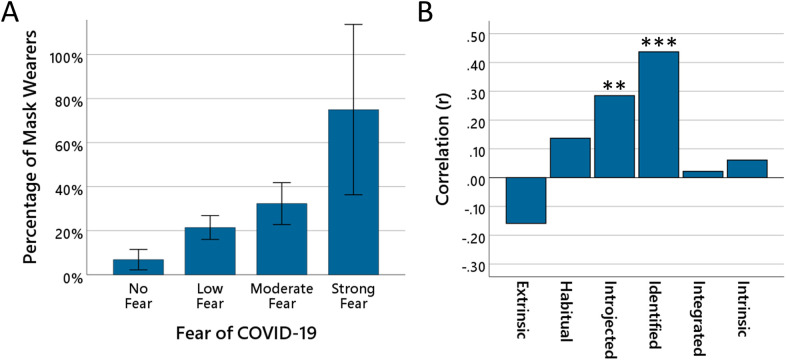
Fear of COVID-19 and internalization of mask wearing. The bars in panel (A) show the proportion of participants (*N* = 445) who reported continuing to wear masks for each level of fear of COVID-19: none, low, moderate, or high. Error bars represent 95% confidence intervals. The bars in (B) show for the group of participants who reported continuing to wear masks (*N* = 93) the size of the correlations between the amount of fear of COVID-19 and the amount of motivation reported at the different levels of internal regulation (extrinsic, habitual, introjected, identified, integrated, and intrinsic motivation).

Pearson correlations among the study variables are reported in S1 Table (Panel B). As shown in Fig 1A, descriptive analyses illustrated a clear positive trend between fear of COVID-19 and continued mask-wearing after the lifting of the mask mandate. The proportion of participants reporting mask use increased with fear level: 6.8% for no fear, 21.4% for slight fear, 32.3% for moderate fear, and 75% for strong fear.

A binary logistic regression showed that fear of COVID-19 significantly predicted continued mask-wearing (see Table 2, Panel A). Because fear was entered as a continuous predictor, the odds ratio reflects the change in odds associated with a one-unit increase on the fear scale. Each one-point increase in fear was associated with a 2.5-fold increase in the odds of continued mask-wearing. This pattern is consistent with the assumption that higher levels of fear of COVID-19 are associated with a greater likelihood of internalizing the previously externally regulated wearing of a mask.

**Table 2:** Logistic regression models predicting continued mask-wearing.

Panel A. Model 1. Fear of COVID-19					
**Predictor**	**B**	**SE**	**p**	**Exp(B)**	**95% CI**
Fear of COVID-19 (centered)	0.916	0.169	<.001	2.50	[1.79–3.48]
*Model fit: −2 Log Likelihood = 424.00; Nagelkerke R² = .071*					
**Panel B. Model 2. Fear of COVID-19 × Mask effectiveness**					
**Predictor**	**B**	**SE**	**p**	**Exp(B)**	**95% CI**
Fear of COVID-19 (centered)	0.864	0.196	<.001	2.37	[1.62–3.48]
Mask effectiveness (centered)	0.212	0.080	.008	1.24	[1.06–1.45]
Fear × Mask effectiveness	−0.135	0.117	.246	0.87	[0.70–1.10]
*Model fit: −2 Log Likelihood = 414.61; Nagelkerke R² = .091*					
**Panel C. Model 3. Fear of COVID-19 × Mask side effects**					
**Predictor**	**B**	**SE**	**p**	**Exp(B)**	**95% CI**
Fear of COVID-19 (centered)	0.922	0.200	<.001	2.51	[1.70–3.72]
Mask side effects (centered)	−0.587	0.179	.001	0.56	[0.39–0.79]
Fear × Mask side effects	0.357	0.243	.143	1.43	[0.89–2.30]
*Model fit: −2 Log Likelihood = 409.88; Nagelkerke R² = .102*					

**Note.** Outcome variable coded 0 = no continued mask-wearing and 1 = continued mask-wearing. Continuous predictors were mean-centered prior to computing interaction terms. Values represent logistic regression coefficients (B), standard errors (SE), odds ratios (Exp(B)), and 95% confidence intervals. N = 445.

To examine whether the association between fear and continued mask-wearing varied depending on participants’ beliefs about the effectiveness of masks in preventing infection, a binary logistic regression was conducted including centered scores of fear of COVID-19, perceived mask effectiveness, and their interaction term as predictors (see Table 2, Panel B). The analysis revealed significant main effects of both fear and perceived mask effectiveness. However, the interaction term was not significant, indicating that the association between fear and continued mask-wearing did not vary as a function of perceived mask effectiveness.

**Fig 2:**
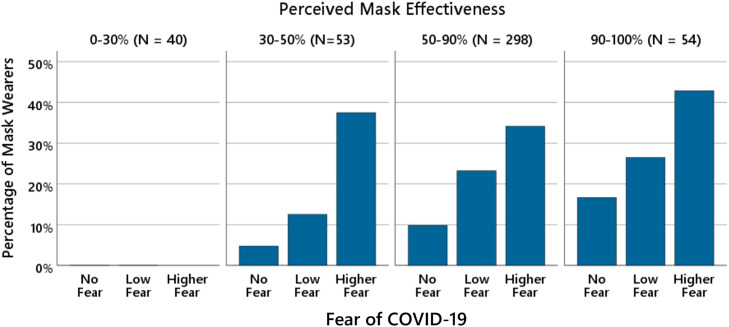
Fear of COVID-19 and continued mask-wearing as a function of perceived mask effectiveness. The bars show the percentage of participants who continued wearing masks as a function of fear of COVID-19 and perceived mask effectiveness (*N* = 445). Participants were divided into four groups according to their perceived mask effectiveness (0–30%, 30–50%, 50–90%, 90–100%). For fear ratings, scores of 3 (“moderate fear”) and 4 (“high fear”) were combined into a single “higher fear” category. Fear categories are displayed for descriptive purposes only; statistical analyses treated fear as a continuous predictor.

To further illustrate the joint effects of fear and perceived mask effectiveness on continued mask-wearing, participants were divided into four groups based on their beliefs about mask effectiveness: low effectiveness (0–30%), somewhat effective (30–50%), moderately effective (50–90%), and highly effective (90–100%). Within each group, the percentage of individuals who continued to wear masks was calculated separately for participants reporting no fear (rating = 1), low fear (rating = 2), and higher fear (ratings = 3 and 4 combined). As shown in Fig 2, the percentage of mask wearers increased with higher levels of fear within each perceived mask effectiveness group.

At first glance, the data suggest a possible threshold effect: in the group perceiving masks as largely ineffective (0–30%), no participant continued to wear a mask. This pattern could indicate that a minimum level of belief in the effectiveness of mask-wearing is necessary for fear to influence behavior. However, it is important to note that this group contained no individuals with higher fear ratings, making it impossible to evaluate the potential effect of fear in this subgroup. Overall, the figure visually corroborates with the main findings of the regression analysis: both higher fear ratings and stronger beliefs in mask effectiveness independently predicted continued mask-wearing, with no evidence of a statistical interaction between these factors.

To examine whether the association between fear and continued mask-wearing varied depending on participants’ beliefs about the strength of side effects associated with mask-wearing, a binary logistic regression was conducted including centered scores of fear of COVID-19, perceived mask side effects, and their interaction term as predictors (see Table 2, Panel C). The analysis revealed significant main effects of both fear and perceived mask side effects. However, the interaction term was not significant, indicating that the association between fear and continued mask-wearing did not vary as a function of perceived mask side effects.

**Fig 3:**
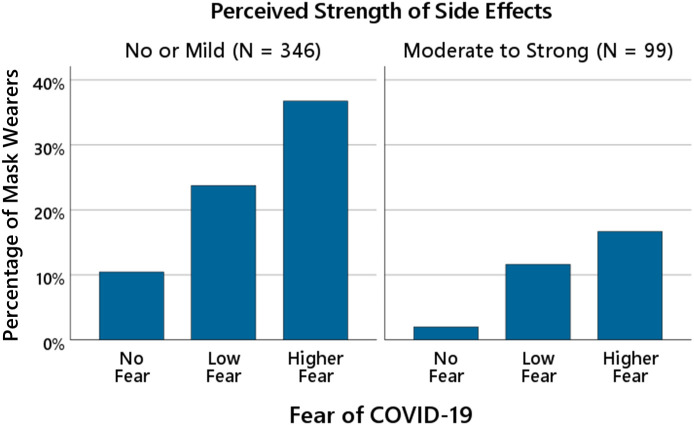
Fear of COVID-19 and continued mask wearing as a function of perceived mask side effects. The bars show the percentage of participants who continued wearing masks as a function of fear of COVID-19 and perceived strength of side effects (*N* = 445). Participants were divided into two groups according to their perceived strength of side effects (no or mild side effects vs. moderate to strong side effects). For fear ratings, scores of 3 (“moderate fear”) and 4 (“high fear”) were combined into a single “higher fear” category. Fear categories are displayed for descriptive purposes only; statistical analyses treated fear as a continuous predictor.

To further illustrate the joint effects of fear and perceived strength of mask side effects on continued mask-wearing, participants were divided into two groups based on their beliefs about the strength of side effects: no or mild side effects (ratings 1 and 2) and moderate to strong side effects (ratings 3, 4, and 5). Within each group, the percentage of individuals who continued to wear masks was calculated separately for participants reporting no fear (rating = 1), low fear (rating = 2), and higher fear (ratings = 3 and 4 combined). As shown in Fig 3, the percentage of mask wearers increased with higher levels of fear within each side effects group.

### Fear of COVID-19 and depth of internalization of mask-wearing

Next, we examined up to which level of internal regulation an effect of fear of COVID-19 could be observed. To this end, correlations were computed between fear of COVID-19 and the amount of motivation reported by participants at the different levels of internal regulation (extrinsic, habitual, introjected, identified, integrated, and intrinsic). The results are displayed in Fig 1B. The strength of the correlation increased from the levels of extrinsic regulation (*r* = –0.16, *p* = 0.129) and habitual regulation (*r* = 0.14, *p* = 0.191) up to the levels of introjected regulation (*r* = 0.285, *p* = 0.006) and identified regulation (*r* = 0.437, *p* < 0.001), where significant associations were observed. At the more internal levels of integrated regulation (r = 0.02, *p* = 0.831) and intrinsic regulation (*r* = 0.06, *p* = 0.563), fear of COVID-19 was no longer associated with motivation.

To examine whether the observed associations between fear ratings and the amount of motivation at the introjected and identified levels of regulation vary depending on participants’ beliefs about the effectiveness and the side effects of mask-wearing, we first computed correlations between beliefs about mask effectiveness and side effects and the amount of motivation at the different levels of internal regulation to identify the levels at which these beliefs were relevant. Belief in mask effectiveness was positively correlated with identified regulation (*r* = .37, *p* < .001), whereas no significant associations were observed with any other levels of regulation (*all ps* > .11). Perceived side effects of mask-wearing were negatively correlated with motivation at the levels of habitual (*r* = –.26, *p* = .011), identified (*r* = –.34, *p* = .001), integrated (*r* = –.32, *p* = .002), and intrinsic (*r* = –.34, *p* < .001) regulation, but not at the level of introjected regulation (*r* = –.10, *p* = .347).

This pattern indicates that both fear and rational beliefs—specifically, belief in mask effectiveness—played a role only at the level of identified regulation. To examine whether fear functions independently of rational beliefs at this level, a linear regression analysis was conducted with fear of COVID-19, belief in mask effectiveness, and their interaction term as predictors of identified regulation. The results revealed significant main effects of both predictors. Higher levels of fear were associated with stronger identified motivation (*B* = 0.32, *SE* = 0.07, *t* = 4.72, *p* < .001), and participants who believed more strongly in the effectiveness of masks also reported higher levels of identified motivation (*B* = 0.11, *SE* = 0.04, *t* = 2.96, *p* = .004). Impor*t*antly, the interaction term was also significant (*B* = –0.14, *SE* = 0.05, *t* = –2.75, *p* = .007). De*t*ailed regression results are reported in S2 Table. This interaction indicates that the association between fear and identified motivation was weaker among participants with stronger beliefs in mask effectiveness.

**Fig 4:**
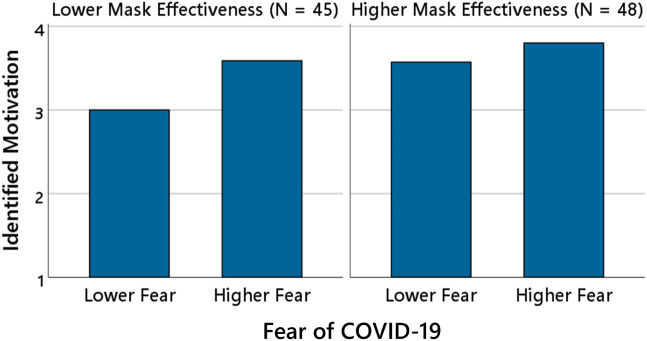
Fear of COVID-19 and identified motivation as a function of perceived mask effectiveness. The bars show the mean level of identified motivation as a function of increasing levels of fear of COVID-19 and perceived mask effectiveness (lower vs. higher; median split). Participants (*N* = 93) were divided according to their perceived mask effectiveness and their reported fear of COVID-19. Fear categories are displayed for descriptive purposes only; statistical analyses treated fear as a continuous predictor.

To illustrate this moderating effect, participants were divided into two groups based on a median split of perceived mask effectiveness (lower vs. higher effectiveness). Within each group, the mean level of identified motivation was calculated separately for participants reporting lower fear (ratings 1 and 2 combined) and higher fear (ratings 3 and 4 combined). As shown in Fig 4, identified motivation increased more strongly with higher fear ratings in the group of participants who believed that masks were less effective. Because participants who believed that masks were highly effective already reported very high levels of identified motivation (*M* = 3.64, *SD* = 0.43), this pattern likely reflects a ceiling effect—indicating that little variance remained to be explained by fear.

### Gender effects

To examine potential gender effects, additional analyses were conducted including gender as a covariate and testing for a possible interaction between gender and fear of COVID-19. In a first model including both fear of COVID-19 and gender as predictors, fear of COVID-19 remained a strong and significant predictor of continued mask-wearing, *B* = 0.90, *SE* = 0.17, *Wald* = 27.65, *p* < .001, *Exp(B)* = 2.46, whereas gender showed no significant effect, *B* = 0.06, *SE* = 0.32, *Wald* = 0.03, *p* = .858, *Exp(B)* = 1.06. In a subsequent model, an interaction term between fear and gender was added to test whether the strength of the association differed between men and women. The interaction term did not reach significance, *B* = 0.73, *SE* = 0.48, *Wald* = 2.31, *p* = .128, *Exp(B)* = 2.07, indicating that the relationship between fear and mask-wearing was similar across genders. Thus, the predictive effect of fear of COVID-19 on continued mask-wearing appears to be robust and not moderated by gender.

## Discussion

The present study took advantage of a unique and ecologically valid opportunity to examine the role of fear in the internalization of external regulation: the introduction and subsequent removal of the mask mandate in Germany, where mask wearing had little prior intrinsic or cultural value and was virtually absent from public life. Specifically, we investigated whether fear of COVID-19 predicted the likelihood of continuing to wear a mask after the mandate was lifted, and up to which level of internalized motivation fear played a role.

The results showed that fear of COVID-19 strongly predicted continued mask-wearing. The logistic regression indicated a 2.5-fold increase in the odds of maintaining the behavior for each one-point increase in fear. This finding suggests that fear can facilitate the internalization of externally regulated behavior and supports the theoretical proposition that the emotional salience of the issue addressed by the externally regulated behavior can energize the internalization process. Notably, the association between fear and internalization was independent of rational beliefs about the effectiveness or the negative side effects of mask-wearing. The effect of fear was present even among individuals who were less convinced of the mask’s effectiveness or who anticipated stronger side effects.

However, analyses of motivational strength among participants who continued to wear masks across different levels of internal regulation revealed that the influence of fear was confined to intermediate stages of internalization. Fear correlated significantly with introjected and identified regulation, but not with integrated or intrinsic regulation. The association between fear and increased introjected motivation aligns well with the assumptions of the Self-Determination Theory [1], which postulates that behavioral regulation at the level of introjection is driven by affective forces such as guilt, anxiety, or fear. This interpretation is further supported by the present finding that rational beliefs—specifically, beliefs about the effectiveness and side effects of mask-wearing—showed no association with motivational strength at this level of regulation.

The finding that fear was also associated with a more deeply internalized form of motivation, namely identified regulation, extends previous research by demonstrating that, under certain conditions, emotions can trigger the cognitive processes necessary for valuing a behavior, thereby moving beyond mere reactive control as seen at the habitual and introjected levels. Moreover, the finding that the effect of fear was stronger among individuals who held weaker beliefs about the effectiveness of mask-wearing suggests that fear may exert its strongest influence when rational conviction is low and cognitive justifications for the behavior are less salient. However, fear does not appear to promote full integration of the behavior into a coherent self-concept. Integrated regulation requires that the behavior one identifies with is harmonized with broader personal values and a sense of self, which appears to be independent of fear.

### Limitations

Several limitations of the present study should be acknowledged. First, the study was exploratory in nature and focused on a subset of variables drawn from a more comprehensive questionnaire assessing a range of factors related to mask-wearing. As such, the analyses were intended to generate hypotheses rather than provide definitive causal explanations. Furthermore, the data are correlational and cross-sectional, which precludes causal inferences about the relationships between fear, rational beliefs, and the internalization of mask-wearing behavior. Future research using longitudinal or experimental designs is needed to clarify the directionality of these effects.

Second, mask-wearing behavior was assessed using a hypothetical scenario—specifically, the situation of visiting a supermarket. Because this scenario represents a common and familiar everyday context, the responses are likely to reflect participants’ actual behavioral tendencies with reasonable validity. However, it cannot be ruled out that, for some individuals, their reported hypothetical behavior may not fully correspond to their real-life actions. Moreover, even within the hypothetical scenario, external regulations may still have played a role despite the official lifting of the mask mandate. For instance, some participants may have anticipated that others present in the supermarket would view not wearing a mask as socially undesirable and might have adjusted their responses accordingly to avoid such external contingencies.

Third, as is typical for many studies investigating the functioning of psychological mechanisms [28], the present sample consisted primarily of university students. Accordingly, the question arises as to whether the observed results can be generalized to other populations. With respect to the proportion of individuals who demonstrated internalization of mask-wearing, it may not be appropriate to assume generalizability to older adults or individuals from other cultural contexts, given evidence that adherence to COVID-19 measures has varied across age groups and countries [29–30]. However, it is important to note, first, that mask-wearing served merely as an example of externally regulated behavior used to study the mechanisms of internalization; thus, the absolute proportion of individuals who had internalized this specific behavior is not directly relevant to the central research question. Second, the finding that the association between fear and internalization of mask-wearing was independent of participants’ gender at least suggests that such demographic factors may not play a major role in the observed relationships. Because the sample consisted primarily of university students, social norms and educational background may have influenced overall compliance levels. However, such influences would be expected to primarily affect externally regulated or socially integrated behavior. In contrast, fear was associated only with intermediate stages of internalization (introjected and identified regulation), but not with integrated regulation. This pattern suggests that the observed relationships cannot be fully explained by socially shared norms within a university context and instead reflect differences in motivational internalization.

## Conclusion

The present findings demonstrate that fear can play an important role in the internalization of externally regulated behaviors by supporting the transition from externally imposed to partially self-determined motivation. However, this effect appears to be limited to intermediate stages of internalization. Fear may help initiate the valuing of a behavior but does not promote its full integration into one’s self-concept. These findings also complement previous research on public health behaviors during the COVID-19 pandemic, which has emphasized the role of autonomous motivation in sustaining compliance [19–21]. While these studies primarily differentiated between controlled and autonomous motivation, the present work provides a more nuanced perspective by showing how emotional factors such as fear, together with rational beliefs about the effectiveness and potential side effects of a behavior, influence motivation at specific stages of internalization.

With regard to public health practice, the present results highlight the need for careful reconsideration of fear-based approaches [31]. While the experience of fear can foster greater internalization, fear-related internalization typically reaches only the level of identified regulation rather than integrated regulation. Consequently, the internalized behavior may not be fully congruent with other internalized or intrinsic behaviors, which can lead to defensiveness or psychological tension. Moreover, since fear was found to promote the internalization of externally regulated behaviors even among individuals who were less convinced of the behavior’s effectiveness or expected stronger negative side effects, these findings indicate that strategies relying on the elicitation of fear must be applied with particular caution if the goal is to foster genuinely internalized, self-determined motivation that aligns with individuals’ rational beliefs and values. Health communication strategies should therefore aim to encourage rational reflection on the advantages and disadvantages of protective behaviors and avoid the unnecessary elicitation of negative emotions.

## Supporting information

S1 Table Descriptive statistics and correlation matrix of the study variables. (DOCX)

S2 Table Linear regression model predicting identified motivation. (DOCX)
