# Supplementary material for: Fear and the internalization of external regulation – An exploratory study on how fear of COVID-19 affected the internalization of mask-wearing
Source: PLoS One. 2026 May 12;21(5):e0347772. doi: 10.1371/journal.pone.0347772 (PMC13166927; doi:10.1371/journal.pone.0347772)
Supplement: S1 Table — (DOCX) [file pone.0347772.s001.docx]

**S1 Table. Descriptive statistics and correlation matrix of the study variables.**

**A. Descriptive statistics**

| **Variable** | **Mean** | **SD** | **Scale** |
| --- | --- | --- | --- |
| Fear of COVID-19 | 1.99 | 0.74 | 1–4 |
| Mask effectiveness | 6.68 | 2.04 | 1–10 |
| Mask side effects | 2.06 | 0.97 | 1–5 |
| External regulation | 2.58 | 0.93 | 1–4 |
| Habitual responding | 2.78 | 0.82 | 1–4 |
| Introjected regulation | 2.98 | 0.86 | 1–4 |
| Identified regulation | 3.06 | 0.92 | 1–4 |
| Integrated regulation | 3.00 | 0.92 | 1–4 |
| Intrinsic regulation | 1.42 | 0.64 | 1–4 |

**B. Correlation matrix**

| **Variable** | **1** | **2** | **3** | **4** | **5** | **6** | **7** | **8** | **9** |
| --- | --- | --- | --- | --- | --- | --- | --- | --- | --- |
| 1 Mask-Wearing | — |  |  |  |  |  |  |  |  |
| 2 Fear of COVID-19 | .27 | — |  |  |  |  |  |  |  |
| 3 Mask effectiveness | .20 | .38 | — |  |  |  |  |  |  |
| 4 Mask side effects | −.21 | −.30 | −.50 | — |  |  |  |  |  |
| 5 External regulation | −.38 | −.27 | −.30 | .18 | — |  |  |  |  |
| 6 Habitual responding | .28 | .31 | .40 | −.44 | −.15 | — |  |  |  |
| 7 Introjected regulation | .11 | .35 | .47 | −.46 | −.02 | .48 | — |  |  |
| 8 Identified regulation | .22 | .46 | .61 | −.58 | −.31 | .47 | .65 | — |  |
| 9 Integrated regulation | .14 | .36 | .58 | −.52 | −.19 | .43 | .64 | .72 | — |
| 10 Intrinsic regulation | .35 | .25 | .22 | −.32 | −.26 | .34 | .27 | .35 | .30 |

**Note.** Values in the correlation matrix represent Pearson correlation coefficients. Mask-wearing was coded 0 = no continued mask-wearing and 1 = continued mask-wearing. N = 445.
