# Supplementary material for: Fear and the internalization of external regulation – An exploratory study on how fear of COVID-19 affected the internalization of mask-wearing
Source: PLoS One. 2026 May 12;21(5):e0347772. doi: 10.1371/journal.pone.0347772 (PMC13166927; doi:10.1371/journal.pone.0347772)
Supplement: S2 Table — (DOCX) [file pone.0347772.s002.docx]

**S2 Table. Linear regression model predicting identified motivation.**

| **Predictor** | **B** | **SE** | **t** | **p** | **95% CI** |
| --- | --- | --- | --- | --- | --- |
| Fear of COVID-19 (centered) | 0.323 | 0.069 | 4.72 | < .001 | [0.19, 0.46] |
| Mask effectiveness (centered) | 0.109 | 0.037 | 2.96 | .004 | [0.04, 0.18] |
| Fear × Mask effectiveness | −0.140 | 0.051 | −2.75 | .007 | [−0.24, −0.04] |

**Model fit**

| **Statistic** | **Value** |
| --- | --- |
| R² | 0.343 |
| Adjusted R² | 0.321 |
| F(3, 89) | 15.52 |
| Model p | < .001 |

**Note**. Outcome variable: identified motivation. Predictors were mean-centered before computing the interaction term. Values represent linear regression coefficients (B), standard errors (SE), t statistics, p values, and 95% confidence intervals. N = 93.
